# Students’ Knowledge about Cervical Cancer Prevention in Poland

**DOI:** 10.3390/medicina57101045

**Published:** 2021-09-30

**Authors:** Karolina Osowiecka, Samira Yahuza, Marek Szwiec, Anna Gwara, Karolina Kasprzycka, Monika Godawska, Dominik Olejniczak, Agnieszka Nowacka, Jacek J. Nowakowski, Sergiusz Nawrocki, Monika Rucinska

**Affiliations:** 1 Department of Psychology and Sociology of Health and Public Health, School of Public Health, University of Warmia and Mazury in Olsztyn, ul. Warszawska 30, 11-041 Olsztyn, Poland; 2 Department of Oncology, University of Warmia and Mazury in Olsztyn, ul. Wojska Polskiego 37, 10-228 Olsztyn, Poland; sami.yahuza@gmail.com (S.Y.); sergiusz.nawrocki@me.com (S.N.); m_rucinska@poczta.onet.pl (M.R.); 3 Department of Surgery and Oncology, Faculty of Medicine and Health Sciences, University of Zielona Gora, ul. Zyty 28, 65-046 Zielona Gora, Poland; szwiec72@gmail.com; 4 Department of Nursing, Institute of Health Science, University of Zielona Gora, ul. Zyty 28, 65-046 Zielona Gora, Poland; a.gwara@poczta.fm; 5 Department of Oncology and Radiotherapy, Faculty of Medical Sciences, Medical University of Silesia in Katowice, ul. Ceglana 35, 40-515 Katowice, Poland; k.kasprzycka@gazeta.pl (K.K.); monikagodawska@hotmail.com (M.G.); 6 Department of Public Health, Medical University of Warsaw, ul. Nielubowicza 5, 02-097 Warsaw, Poland; dominikolejniczak@op.pl; 7 Department of Gynecology and Obstetrics Didactics, Faculty of Health Sciences, Medical University of Warsaw, ul. Litewska 14/16, 00-575 Warsaw, Poland; agnieszka.nowacka@wum.edu.pl; 8 Department of Ecology and Environmental Protection, Faculty of Biology and Biotechnology, University of Warmia and Mazury in Olsztyn, Plack Łódzki 3, 10-727 Olsztyn, Poland; jacek.nowakowski@uwm.edu.pl

**Keywords:** cervical cancer, HPV, knowledge, primary and secondary prevention

## Abstract

*Background and Objectives*: In Poland, the rates of morbidity and mortality due to cervical cancer are amongst the highest in Europe. A significant percentage of newly diagnosed cases of cervical cancer are at an advanced stage. Unfortunately, only about 20% of Polish women take part in cervical cancer screening. The aim of the study was to assess students’ knowledge of cervical cancer risk factors and prevention. *Materials and Methods*: The study was provided to Polish students from various universities and faculties between May 2020 and November 2020. The questionnaire was designed specifically for this study and was validated. The chi-square test was used to compare the responses between subgroups. *Results*: The study was carried out on a group of 995 students (80.6% women, 19% men, 0.4% no data), (average age 21.9 years). Most students knew that the main risk factor for cervical cancer is human papillomavirus (HPV) infection (82% of all responders; 86% of medical students; 73% of non-medical students; *p* < 0.001). Only 40% of students knew that in Poland the Population Prevention and Early Diagnosis Program is carried out on women aged 25–59 years every three years. Most students correctly indicated that cervical cancer screening in Poland is performed using cervical cytology and were familiar with the basis of cytology. Only 57% of students knew that there are no specific early symptoms of cervical cancer. A total of 78% of all respondents knew that HPV vaccination reduces the risk of cervical cancer. Medical students and students who are sexually active demonstrated a better knowledge of cervical cancer. *Conclusions*: The Polish students had some knowledge of cervical cancer risk factors and primary and secondary prevention. Significantly better knowledge was demonstrated by medical students. Some efforts should be made to ensure that young people, who are not associated with medicine are better educated about cervical cancer in order to reduce the overall incidence and improve early detection rates.

## 1. Introduction

Cervical cancer remains a serious problem although the prevalence of cervical cancer has decreased in developed countries in recent years. The estimated number of new cervical cancer cases in 2020 worldwide was over 600 thousand (3.1% of all cancers in women) [1]. In Poland, 2360 new cases and 1593 deaths were reported in 2018 [2]. The rates of morbidity and mortality due to cervical cancer in Poland are amongst the highest in Europe: The crude rates of incidence are 19.5 vs. 13.3 and of mortality 10.8 vs. 5.9, respectively [3]. Although the standardized rate of deaths from cervical cancer has been systematically reduced in Poland during last twenty years (from 8.74 in 1999 to 5.57 in 2018) [2], the five-year relative survival rate of women diagnosed with cervical cancer in the period 2000–2007 was 55.2–10% less than the European average (65.4%) [4]. This is probably because 40% of new cases of cervical cancer in Polish women are diagnosed at a more advanced stage [2].

Human papillomavirus (HPV) infection has been identified as a prerequisite for the development of cervical cancer [5]. It is one of the most prevalent sexually transmitted infections in the world. About 50–80% of sexually active women acquire infection by at least one type of HPV during their lifetime [5,6]. Most cases of cervical cancer are caused by HPV type 16 and 18 [7]. The highest prevalence rates of HPV infections are observed in young women aged about 25 years [8,9]. Primary prevention of cervical cancer is related to the prevention of HPV infection. Since 2006 a vaccine against HPV has been available. The immunization programs against HPV were recommended by the World Health Organization (WHO) [10]. As of June 2020, 107 of the 194 WHO Member States (55%) had introduced national HPV vaccination programs. The Americas and Europe have the highest rates of national vaccination programs (85% and 77% of countries, respectively) whereas in Oceania, Asia and Africa the coverage rates are: 56%, 40% and 31% of countries [11]. Unfortunately, in Poland (similar to some other European countries, e.g., Cyprus, Estonia, Finland, Hungary, Lithuania, Malta, and Slovakia) there is no vaccination program against the HPV. According to the Polish National Oncological Strategy a vaccination program against papillomavirus should be introduced in 2021 for girls and in 2026 for boys. By 2028 it is expected that at least 60% of girls and boys will be vaccinated against the human papillomavirus in Poland [12,13].

Secondary prevention of cervical cancer involves cytological screening. The WHO suggests using HPV DNA detection screening at the age of 30 years with regular screening every five to 10 years [14]. In the United States, there are three possible screening strategies recommended; screening every three years with cytology alone, screening with HPV testing alone every five years or using HPV testing in combination with cytology every five years in women 30–65 years [15,16,17,18]. The European countries which have implemented national HPV-based screening are: the Netherlands, Turkey, Italy, Sweden, Finland, Spain, Norway, Denmark, United Kingdom, Belgium, Germany and Malta [19,20,21,22,23,24,25,26,27,28,29,30]. In Poland a pap test every three years is used for cervical cancer screening in women aged 25–59 years. Unfortunately, only about 20% of Polish women take part in this screening [2]. One characteristic of cervical cancer secondary prevention in Poland is the high percentage of cytological tests performed outside the public health care system. In 2014, 85% of women declared that they had undergone a pap smear as part of private diagnostics at least once, and about 30% of women indicated that the test had taken place within the last year [31]. The number of tests performed outside the system financed from public funds is not reported. There is a possibility that some women undergo tests more often than is recommended, while in other groups, especially those with a lower socioeconomic status, women may take part in tests less often than recommended or are not tested at all [32].

One of the reasons women do not participate in screening is a lack of knowledge about cervical cancer and its prevention [33,34,35]. Additionally, a poor awareness of HPV and the association between HPV and cervical cancer was observed [36,37,38,39,40,41]. Therefore, it could be a good idea to improve public awareness about cervical cancer issue. Especially the level of knowledge about primary and secondary prevention amongst young people should be checked to facilitate appropriate educational planning.

The aim of the study was to assess Polish students’ knowledge of cervical cancer risk factors and prevention.

## 2. Materials and Methods

### 2.1. Study Design and Questionnaire

The study was conducted among 995 Polish students from various universities and faculties between May 2020 and November 2020.

The questionnaire was designed specifically for this study in accordance with general principles [42,43]. The questionnaire consisted of 8 main closed-ended quantitative questions and 6 questions concerning demographic data (age, gender, faculty of study, degree of study, sexual activity, a conversation about cervical cancer) (see Supplementary Materials). The comprehensibility and acceptability of the questionnaire was validated by a psycho-oncologist. The pilot study was conducted on a group of 49 medical students. After the first twenty students filled out the questionnaire, they were interviewed to verify they properly understood the questions. The questionnaire was validated on a group of 62 students. In the validation procedure, a reliability analysis of the questionnaire was used—the comparison of two responses to questions from the same questionnaire from the same person, over an interval of time (i.e., an estimation of the absolute stability of the questionnaire). An estimation of the degree of reproducibility of individual responses was made using the Kappa-Cohen’s coefficient of agreement [44]. The values of coefficients were tested with the Kappa-Cohen significance test used to verify the hypothesis value kappa = 0, indicating the randomness of the results of double measurements of responses. Kappa-Cohen’s coefficient describes the degree of agreement of duplicate measurements of the same variable; is determined for dependent categorical variables, and its value ranges from −1 to 1, where the value 1 means full compliance, and the value 0 means compliance at a random level. In this study, to interpret the result of the statistical analysis of repeatability, a conventional scale was used, assigning the following meaning to individual values of the Kappa-Cohen’s coefficient—0.81–1.00 very good repeatability, 0.61–0.80 good repeatability, 0.41–0.60 moderate repeatability, 0.20–0.40 poor repeatability, <0.21 very poor repeatability [45]. The compatibility of the answers was also tested with the Cramér V coefficient, which determines the level of dependence between two nominal variables, of which at least one has more than two categories [46]. Cramér’s V coefficient results in values between 0 and 1, and the closer the score is to 1, the stronger the relationship between the responses indicated in the first and second completion of the questionnaire. The questionnaire was completed via the Internet or in paper form. Participation in the study was voluntary and anonymous. By starting to fill in the questionnaire, the students gave their consent to participation in the study.

The study protocol was approved by the Ethics Committee of the University of Warmia and Mazury in Olsztyn (No. 12/2020).

For all questions, the significance test indicated that Kappa-Cohen’s coefficient was significantly different from zero, suggesting that the measurements were consistent (repeatable). In the case of one question (1) there was very good repeatability, in the case of five (3, 4, 5, 6, 8) good repeatability, and in the case of two (2, 7) moderate repeatability. The Cramer’s V coefficient in all cases was significant different to zero, and indicated the high-to-moderate relationship between responses (Table 1).

### 2.2. Statistical Analysis

The data were calculated using descriptive statistics. Questions were grouped into true and false. The chi-square test was used to compare the proportion between the subgroups. The age differences between responses to each question were assessed using the Mann-Whitney test. A *p* value <0.05 was considered to be significant. The analysis was conducted using Statistica (data analysis software system), version 13. http://statistica.io (accessed on 1 April 2020) TIBCO Software Inc., Krakow, Poland (2017). The Kappa-Cohen’s coefficient, Kappa-Cohen consistency test and Cramer’s V coefficient were calculated using the SPSS 27.0 program (IBM Corp. Released 2020. IBM SPSS Statistics for Windows, Version 27.0. IBM Corp., Armonk, NY, USA).

## 3. Results

The study was conducted on a group of 995 students in the age range 18–28 years old (average age 21.9 ± 1.8 years). The analysis included 802 women (80.6%) and 189 men (19%) (four students—no data). The students were from various Universities in different regions of Poland. More students were from various medical faculties (67.8%) including Medicine, Health Sciences, Veterinary Medicine Faculty. Half of respondents (50.7%) were undertaking a master’s degree. The majority of students (67.3%) indicated that they were sexually active. Only half of students (56.5%) had ever a conversation with someone about cervical cancer (Table 2).

### 3.1. Knowledge about Risk Factors for Cervical Cancer

Most students knew that the main risk factor for cervical cancer is human papillomavirus (HPV) infection (82% of all responders; 86% of medical students vs. 73% of non-medical students; *p* < 0.001). Students of the Medicine Faculty had the greatest knowledge, whereas students from the Theology Faculty had the poorest knowledge about the main risk factor for cancer of the cervix (92.8% vs. 58.3%, respectively; *p* < 0.001). Both older age and higher degree of study were associated with better awareness in respondents (*p* < 0.001). Students undertaking master’s studies significantly more often indicated the correct answer than students undertaking bachelor’s degree (87.7% vs. 75.6%, respectively). There were no differences observed between respondents of different genders, based on sexual activity or having had a conversation with someone about cervical cancer (*p* > 0.05).

78% of all respondents knew that HPV vaccination reduces the risk of cervical cancer (83% of medical students vs. 67% of non-medical students; 80% of women vs. 71% of men; 81.5% of students during master’s degree vs. 74% of students during bachelor’s degree; *p* < 0.05). Only 41.7% of students from Theology Faculty indicated the HPV vaccine as a method of reducing the incidence of cervical cancer.

### 3.2. Knowledge about Cervical Cancer Screening

In Poland, the Population Prevention and Early Diagnosis Program involves screening of women age 25–59 years every three years. Only 40% of all students had this knowledge. Although there was no significant difference between medical and non-medical students, the difference was significant between faculties. The respondents from Medicine Faculty were most aware (50.7%), whereas Veterinary Medicine and Humanistic Faculty students were the least aware (about 20%). Older age and higher degree of study were also factors correlated with knowledge of the Population Prevention and Early Diagnosis Program (*p* < 0.001). There was no significant difference in knowledge of the Population Prevention and Early Diagnosis Program when correlated with other respondent characteristics (gender, sexual activity, having a conversation about cervical cancer).

Students correctly indicated that cervical cancer screening in Poland is performed using cervical cytology (87%), significantly often older, women (90%), medical students (93%), during master’s degree (89%) and responders sexually active (88.8%) (*p* < 0.05). The correct answer was most frequently indicated by students of Medicine, Health Sciences and Veterinary Medicine Faculty (about 90%), whereas only 41.7% of responses from students of Theology Faculty were correct.

A total of 71% of all students were familiar with basis of cytology (77% of medical students vs. 58% of non-medical students). The most knowledgeable in this regard were students of Medicine Faculty (87%) and the least knowledgeable were students of Theology (33%) and students of Humanistic Faculty (41%) (*p* < 0.001). Older age and higher degree of study significantly correlated with better knowledge, whereas a comparison of respondents based on sex or sexual activity showed no significant difference for this question.

A total of 80% of respondents claimed that the first preventive cytological examination should be done shortly after initiation of sexual activity. Significantly more awareness was observed in respondents of older age, women (82%), those who were sexually active (82%), undertaking a master’s degree (86%) and medical students (85%).

### 3.3. Knowledge about Cervical Cancer

Only about half of students (57%) knew that there are no specific early symptoms of cervical cancer (this was more often the case for medical students—60.6% of responders vs. 51.7% of non-medical students, students undertaking a master’s degree—62.9% of responders vs. 52.3% of students undertaking a bachelor’s degree, and responders who were sexually active—60.3% of responders vs. 52.5% of students sexually non-active; *p* < 0.05). The majority of students from Medicine Faculty (66%) indicated correctly that there are no specific early symptoms of cervical cancer, but only about 41% of students from Humanistic or Theology Faculty correctly identified this (*p* = 0.03).

Less than half of students (45%) indicated that cervical cancer is not genetically inherited. A significant difference between groups was noted only based on study faculty. Medical students more frequently responded correctly in comparison to non-medical students (49% vs. 39%; *p* = 0.004).

**Figure 1:**
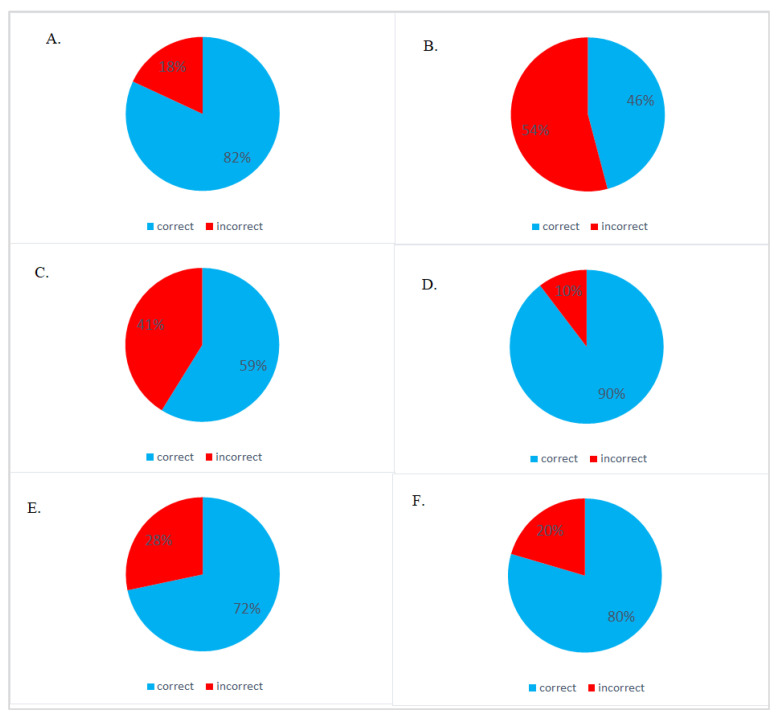
Women respondents’ awareness of risk factor and prevention of cervical cancer. (**A**) What is the main factor risk of cervical cancer? (correct answer: HPV); (**B**) Is cervical cancer genetically inherited? (correct answer: No); (**C**) What is the characteristic symptom of early stage cervical cancer? (correct answer: There are no characteristic symptoms of cervical cancer); (**D**) What is the name of the screening test for cervical cancer? (correct answer: Pap test); (**E**) What is the screening test for cervical cancer? (correct answer: Microscopic evaluation of the exfoliated cells from the vaginal part of the cervix); (**F**) What is the most important for reducing the own risk of cervical cancer? (correct answer: Vaccination against HPV viruses).

**Figure 2:**
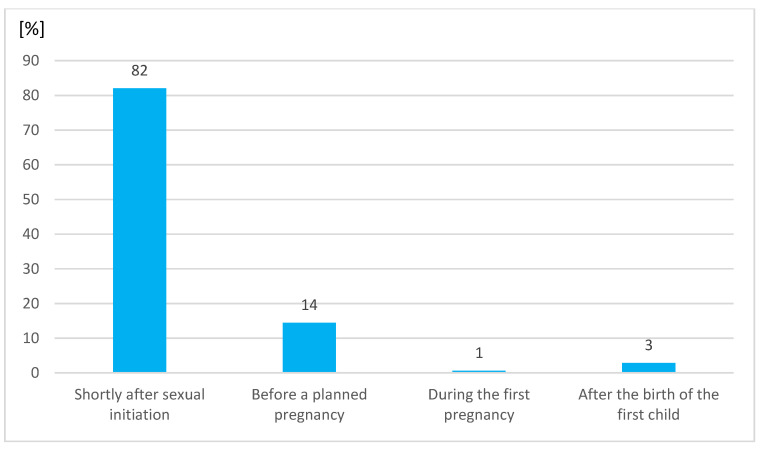
The women’s answers about the point of life when the first prophylactic test for cervical cancer should be done.

The frequency of correct answers for most of the questions did not depend on sex except for the name and age of screening and primary prophylaxis. Only women are afflicted by cervical cancer; therefore, females’ knowledge was analyzed separately (Figure 1 and Figure 2).

## 4. Discussion

Cervical cancer is characteristic due to high worldwide incidence and mortality. However, this cancer could be significantly reduced in the population by early prevention. There is a well-known cervical cancer risk factor—HPV infection. Vaccination against HPV in girls and young women is a proper primary prevention of cervical cancer. In the case of diagnosis at an early clinical stage there are effective treatment methods. Using screening tests it is even possible to detect very early and precancerous stages of cervical cancer. Adequate primary and secondary cervical cancer prophylaxis leads to significant reduction in mortality. For this reason, women should understand the problem and participate in prophylactics programs.

Cervical cancer could affect young women. Therefore we directed our questionnaire on the risk factor and prophylaxis methods for cervical cancer to students. The majority of students knew that HPV infection is the main risk factor for cancer development (82%) and that this risk could be reduce by vaccination (78%). However, it is surprising that about half of them claimed that there is relationship between the development of cervical cancer and genetic inheritance. Another Polish study of 149 female students, with an average age of 20 years, at the University in Kielce also showed that 94% of students were familiar with the HPV infection [47]. Depending on country, the percentage of students who know that genital HPV infection can cause cervical cancer varied between 20% in Nigeria, through 34% in Costa Rica, 45% in Scotland, 58% in Turkey, to 65% for medical students in China [48,49,50,51,52]. Less than 50% of vocational school students in Germany understood that HPV infection is sexually transmitted [53]. Adults from the US, UK and Australia demonstrated low levels of detailed knowledge about HPV infection and links to cervical cancer. Only 61% of those surveyed had heard about HPV [54]. Young adults’ knowledge about HPV vaccination is insufficient. Among female students from Serbia, only 14% had heard about both the relationship between HPV infection and cervical cancer and the preventive role of a vaccine against HPV on cervical cancer incidence [55]. The best level of student’ knowledge was observed in Belgium and Spain (about 85%) [56]. The highest knowledge among adults about HPV vaccination was noted in UK (90%) and Spain (80%) whereas a low awareness was found in the Netherlands (6%) [56]. A large percentage of participants in our study knew about the preventive role of HPV vaccination, although there is no national vaccination program in Poland. It seems that young women who have been vaccinated may have increased knowledge of HPV and its association with cervical cancer, because the vaccination is accompanied by information from trained school nurses or in the form of leaflets [57].

In our study, medical students were significantly more aware of cervical cancer prevention and risk factors in comparison with non-medical students. Rančić et al. [55] conducted similar research among female students in Serbia and also showed that medical students had significantly higher knowledge about cervical cancer and HPV infection compared to non-medical students. Additionally, students in a relationship and sexually active were more aware; however, we have not observed any correlation between the knowledge about cervical cancer and having sexual intercourse.

Students undertaking master’s degrees in our study had a greater knowledge than those studying for bachelor’s degrees. Similarly, Marlow’s study [54] showed the relationship between higher education and better knowledge. Female students appeared to know more than male students about HPV infection and HPV vaccination [50,52]. Additionally, in an adult group, women had more adequate information [54]. Our results showed that girls had better knowledge about HPV vaccination and the name and age of screening than boys.

It seems that general knowledge about cervical cancer and its primary and secondary prevention are not sufficient. People should be educated about the need for vaccine against HPV and the screening procedure. Knowledge is important for vaccine acceptance [58]. Rodriguez et al. [59] made a meta-analysis including 17 articles involving over 68 thousand children, adolescents and young adults. The meta-analysis indicated that behavioral and informational interventions were effective for HPV vaccine with doubled vaccine initiation. The association between individual knowledge and acceptance and initiation of vaccination against HPV was demonstrated [59]. Education should be directed to young people both girls and boys. Primary prevention of cervical cancer should include their parents passing on safe sexual behaviors to their children and promote vaccination [56]. People should have the possibility to take part in national immunization and screening programs. In some countries HPV vaccine is received due to parental concerns. Parents would like to have more information about HPV before making decisions about their children’s vaccinations [60]. Adolescents with a vaccinated older sister had better knowledge about HPV [56]. In our study, only 56.5% of respondents had participated in a conversation with someone about cervical cancer.

There are gaps in knowledge. About half of the students in our study did not know that there are no specific early symptoms of cervical cancer. The general public’s belief is that if there are no symptoms of disease, the disease does not exist. In case of cervical cancer, women should not wait for symptoms. In the absence of early symptoms, abnormality has to be identified by another method like a screening test. The awareness of screening tests for cervical cancer is crucial among women. The pap test for screening is commonly used across the world. Most of the women in our study knew what kind of examination is used as a screening test for cervical cancer. Only 40% of all investigated students knew that the Population Prevention and Early Diagnosis Program in Poland is directed at women aged 25–59 years and carried out every three years. The average age of students was 22 years and women soon should start participation in the screening program. Unfortunately they were not aware of that. In the study of Marlow et al. [61], 28% of women (aged 25–64 years) who did not participate in cervical cancer screening declared that they were not familiar with screening, especially those of a younger age. A systematic personal invitation for cervical cancer screening seems to be an effective form of information about screening. The one reminder letter used in Lithuania more than doubled the coverage of screening [62].

According to WHO recommendations, it is possible to eliminate cervical cancer if, in a country, 90% of girls are fully vaccinated against HPV by the age of 15, 70% of women are screened using a high-performance test by the age of 35, and again by the age of 45, and 90% of women with cervical cancer receive treatment. WHO estimated that median cervical cancer incidence can be reduced by 10% by 2030 and in a 100-year period 62 million cervical cancer deaths could be avoided [63].

Therefore, young women should be educated about cervical cancer risk factors and be encouraged to undertake primary preventive behaviors (contraception, vaccinations, less sexual partners). Some public initiatives, health programs and TV advertisements could improve uptake in screening tests among young women. However, although cervical cancer affects women, the interventions could be designed to engage men as well. They should also be aware that they could transmit HPV infection. Nowadays, different sexual behaviors could be a reason for development not only of cervical cancer but also oral and rectal cancer. It is important to educate young adults about cancer and primary and secondary prevention methods.

## 5. Conclusions

Although students were aware of the link between HPV and cervical cancer and that the vaccine against HPV reduces a risk of cervical cancer, a few gaps in Polish students’ knowledge were observed. There were significant differences in awareness of cervical cancer prevention between medical and non-medical students. Therefore all young women should be educated in this issue to be able to protect themselves from cervical cancer.

## Figures and Tables

**Table 1:** Kappa-Cohen’s and Cramer V coefficient describing the results for validation of the repeatability of responses.

Question	Kappa-Cohen	SE_kappa_	Test Z	*p*	Cramer’s V	*p*
1	0.895	0.059	8.442	<0.001	0.880	<0.001
2	0.563	0.095	4.781	<0.001	0.607	<0.001
3	0.691	0.084	6.939	<0.001	0.762	<0.001
4	0.789	0.144	8.875	<0.001	0.846	<0.001
5	0.642	0.088	6.289	<0.001	0.518	<0.001
6	0.696	0.100	7.019	<0.001	0.660	<0.001
7	0.575	0.091	6.097	<0.001	0.603	<0.001
8	0.610	0.109	7.005	<0.001	0.620	<0.001

SE_kappa_—asymptotic standard error of Kappa-Cohen’s coefficient.

**Table 2:** Characteristics of respondents.

		*n*	%
Gender			
	Women	802	80.6
	Men	189	19.0
	No data	4	0.4
Faculty			
	Medicine	306	30.8
	Health Sciences	348	35.0
	Veterinary Medicine	20	2.0
	Technical Sciences	89	8.9
	Law/Administration	30	3.0
	Art	2	0.2
	Humanistic	34	3.4
	Social Sciences	50	5.0
	Theology	12	1.2
	Other	104	10.5
Degree of study			
	Bachelor degree	491	49.3
	Master degree	504	50.7
Do you have sexual intercourse?			
	Yes	670	67.3
	No	317	31.9
	No data	8	0.8
Have you ever had a conversation with someone about cervical cancer?			
	Yes	562	56.5
	No	425	42.7
	No data	8	0.8

## Data Availability

All data is available at the Department of Psychology and Sociology of Health and Public Health at University of Warmia and Mazury in Olsztyn, Poland.

## Supplementary Materials

The following are available online at https://www.mdpi.com/article/10.3390/medicina57101045/s1, File1: Example of the questionnaire.

## References

[1] World Health Organization. https://gco.iarc.fr/today/home.

[2] National Cancer Registry. http://onkologia.org.pl/raporty/.

[3] ECIS–European Cancer Information System. https://ecis.jrc.ec.europa.eu/.

[4] Eurocare 5 Survival Analysis 2000–2007. https://w3.iss.it/site/EU5Results/forms/SA0007.aspx.

[5] Keam S.J., Harper D.M. (2008). Human papillomavirus types 16 and 18 vaccine (recombinant, AS04 adjuvanted, adsorbed) [CervarixTM]. BioDrugs.

[6] Baseman J.G., Koutsky L.A. (2005). The epidemiology of human papillomavirus infections. J. Clin. Virol..

[7] Clifford G., Franceschi S., Diaz M., Muñoz N., Villa L.L. (2006). Chapter 3: HPV type-distribution in women with and without cervical neoplastic diseases. Vaccine.

[8] Schiffman M., Castle P.E. (2005). The promise of global cervical cancer prevention. N. Engl. J. Med..

[9] Stanley M. (2010). Pathology and epidemiology of HPV infection in females. Gynecol. Oncol..

[10] World Health Organization (2019). Meeting of the Strategic Advisory Group of Experts on Immunization, April 2019–Conclusions and recommendations. Wkly. Epidemiol. Rec..

[11] Bruni L., Saura-Lázaro A., Montoliu A., Brotons M., Alemany L., Saliou Diallo M., Zeren Afsar O., LaMontagne D.S., Mosina L., Contreras M. (2021). HPV vaccination introduction worldwide and WHO and UNICEF estimates of national HPV immunization coverage 2010–2019. Prev. Med..

[12] European Centre for Disease Prevention and Control Introduction of HPV Vaccines in EU Countries–An Update. https://www.ecdc.europa.eu/sites/default/files/media/en/publications/Publications/20120905_GUI_HPV_vaccine_update.pdf.

[13] Uchwała nr 10 Rady Ministrów z Dnia 4 Lutego 2020 r. w Sprawie Przyjęcia Programu Wieloletniego pn. Narodowa Strategia Onkologiczna na Lata 2020–2030, Monitor Polski, 18 lutego 2020 poz. 189. http://isap.sejm.gov.pl/isap.nsf/DocDetails.xsp?id=WMP20200000189.

[14] World Health Organization New Recommendations for Screening and Treatment to Prevent Cervical Cancer. https://www.who.int/news/item/06-07-2021-new-recommendations-for-screening-and-treatment-to-prevent-cervical-cancer.

[15] Ronco G., Dillner J., Elfström K.M., Tunesi S., Snijders P.J., Arbyn M., Kitchener H., Segnan N., Gilham C., Giorgi-Rossi P. (2014). Efficacy of HPV-based screening for prevention of invasive cervical cancer: Follow-up of four European randomised controlled trials. Lancet.

[16] Von Karsa L., Arbyn M., DeVuyst H., Dillner J., Dillner L., Franceschi S., Patnick J., Ronco G., Segnan N., Suonio E. (2015). European guidelines for quality assurance in cervical cancer screening. Summary of the supplements on HPV screening and vaccination. Papillomavirus Res..

[17] Dijkstra M.G., van Zummeren M., Rozendaal L., van Kemenade F.J., Helmerhorst T.J., Snijders P.J., Meijer C.J., Berkhof J. (2016). Safety of extending screening intervals beyond five years in cervical screening programmes with testing for high risk human papillomavirus: 14 year follow-up of population based randomised cohort in the Netherlands. BMJ.

[18] Curry S.J., Krist A.H., Owens D.K., Barry M.J., Caughey A.B., Davidson K.W., Doubeni C.A., Epling J.W., Kemper A.R., US Preventive Services Task Force (2018). Screening for cervical cancer: US preventive services task force recommendation statement. JAMA.

[19] Polman N.J., Snijders P.J.F., Kenter G.G., Berkhof J., Meijer C.J.L.M. (2019). HPV-based cervical screening: Rationale, expectations and future perspectives of the new Dutch screening programme. Prev. Med..

[20] Gultekin M., Zayifoglu Karaca M., Kucukyildiz I., Dundar S., Boztas G., Semra Turan H., Hacikamiloglu E., Murtuza K., Keskinkilic B., Sencan I. (2018). Initial results of population based cervical cancer screening program using HPV testing in one million Turkish women. Int. J. Cancer.

[21] Gultekin M., Karaca M.Z., Kucukyildiz I., Dundar S., Keskinkilic B., Turkyilmaz M. (2019). Mega HPV laboratories for cervical cancer control: Challenges and recommendations from a case study of Turkey. Papillomavirus Res..

[22] Ronco G., Zappa M., Franceschi S., Tunesi S., Caprioglio A., Confortini M., Del Mistro A., Carozzi F.M., Segnan N., Zorzi M. (2016). Impact of variations in triage cytology interpretation on human papillomavirus-based cervical screening and implications for screening algorithms. Eur. J. Cancer.

[23] Duodecim Current Care Guidelines. https://www.duodecim.fi/english/products/current-care-guidelines/.

[24] Torné Bladé A., Del Pino Saladrigues M., Cusidó Gimferrer M., Alameda Quitllet F., Andia Ortiz D., Castellsagué Piqué X., Cortés Bordoy J., Granados Carreño R., Guarch Troyas R.M., Belén LLoveras R. (2014). Guía de cribado del cáncer de cuello de útero en España, 2014. Prog. Obstet. Ginecol..

[25] Marzo-Castillejo M., Vela-Vallespín C., Bellas-Beceiro B., Bartolomé-Moreno C., Melús-Palazón E., Vilarrubí-Estrella M., Nuin-Villanueva M. (2018). Recomendaciones de prevención del cáncer. Actualización PAPPS 2018. Aten. Primaria.

[26] Tropé A., Engesæter B., Nygård M., Andreassen T., Lönnberg S., Ursin G. (2017). Safe implementation of HPV testing in the Norwegian Cervical Cancer Screening Programme. Tidsskr Nor Laegeforen.

[27] Screening for Livmoderhalskræft. https://www.cancer.dk/forebyg/screening/livmoderhalskraeft/.

[28] Rebolj M., Rimmer J., Denton K., Tidy J., Mathews C., Ellis K., Smith J., Evans C., Giles T., Frew V. (2019). Primary cervical screening with high risk human papillomavirus testing: Observational study. BMJ.

[29] Gemeinsamer Bundesausschuss. https://www.g-ba.de/.

[30] Bruni L., Albero G., Serrano B., Mena M., Gómez D., Muñoz J., Bosch F.X., de Sanjosé S. ICO/IARC Information Centre on HPV and Cancer (HPV Information Centre). Human Papillomavirus and Related Diseases in Malta. Summary Report 17 June 2019. https://hpvcentre.net/statistics/reports/MLT.pdf.

[31] Piekarzewska M., Wieczorkowski R., Zajenkowska-Kozłowska A. Stan Zdrowia Ludności Polski w 2014 r. Główny Urząd Statystyczny. Warszawa 2016. https://stat.gov.pl/obszary-tematyczne/zdrowie/zdrowie/stan-zdrowia-ludnosci-polski-w-2014-r-,6,6.html.

[32] Nowakowski A., Cybulski M., Śliwczyński A., Chil A., Teter Z., Seroczyński P., Arbyn M., Anttila A. (2015). The implementation of an organised cervical screening programme in Poland: An analysis of the adherence to European guidelines. BMC Cancer.

[33] Cunningham M.S., Skrastins E., Fitzpatrick R., Jindal P., Oneko O., Yeates K., Booth C.M., Carpenter J., Aronson K.J. (2015). Cervical cancer screening and HPV vaccine acceptability among rural and urban women in Kilimanjaro Region, Tanzania. BMJ Open.

[34] Maree J.E., Lu X.M., Wright S.C. (2012). Cervical cancer: South African women’s knowledge, lifestyle risks and screening practices. Afr. J. Nurs. Midwifery.

[35] Ndejjo R., Mukama T., Musabyimana A., Musoke D. (2016). Uptake of cervical Cancer screening and associated factors among women in rural Uganda: A cross sectional study. PLoS ONE.

[36] Trim K., Nagji N., Elit L., Roy K. (2011). Parental knowledge, attitudes and behaviours towards human papillomavirus vaccination for their children: A systematic review from 2001 to 2011. Obs. Gynecol. Int..

[37] Cuschieri K., Horne A., Szarewski A., Cubie H.A. (2006). Public awareness of human papillomavirus. J. Med. Screen.

[38] Marlow L.A.V., Waller J., Wardle J. (2007). Public awareness that HPV is a risk factor for cervical cancer. Br. J. Cancer.

[39] Klug S.J., Hukelmann M., Blettner M. (2008). Knowledge about infection with human papillomavirus: A systematic review. Prev. Med..

[40] Tiro J.A., Meissner H.I., Kobrin S., Chollette V. (2006). What do women in the U.S. know about human papillomavirus and cervical cancer?. Cancer Epiedmiol. Biomark. Prev..

[41] Pitts M., Clarke T. (2002). Human papillomavirus infections and risks of cervical cancer: What do women know?. Health Educ. Res..

[42] Burgess T.F. (2001). A General Introduction to the Design of Questionnaires for Survey Research. Guide to the Design of Questionnaires.

[43] Boparai J.K., Singh S., Kathuria P. (2018). How to Design and Validate a Questionnaire: A Guide. Curr. Clin. Pharmacol..

[44] Cohen J. (1960). A coefficient of agreement for nominal scales. Educ. Psychol. Meas..

[45] Brzeziński J. (2003). Metodologia Badań Psychologicznych.

[46] Sheskin D. (2011). Handbook of Parametric and Nonparametric Statistical Procedures.

[47] Lewitowicz P., Horecka-Lewitowicz A., Adamczyk-Gruszka O. (2013). Knowledge of cervical cancer risk factors among students at the Jan Kochanowski University in Kielce. Studia Med..

[48] Carlson L.M., Gonzalez S. (2014). Knowledge of cervical cancer pathology of high school students in San Carlos, Costa Rica. Rev. Biol. Trop..

[49] Rathfisch G., Gungor I., Uzun E. (2015). Human papillomavirus vaccines and cervical cancer: Awareness, knowledge, and risk perception among Turkish undergraduate students. J. Cancer Educ..

[50] McCusker S.M., Macqueen I., Lough G., Macdonald A.I., Campbell C., Graham S.V. (2013). Gaps in detailed knowledge of human papillomavirus (HPV) and the HPV vaccine among medical students in Scotland. BMC Public Health.

[51] Makwe C.C., Anorlu R.I., Odeyemi K.A. (2012). Human papillomavirus (HPV) infection and vaccines: Knowledge, attitude and perception among female students at the University of Lagos, Lagos, Nigeria. J. Epidemiol. Glob. Health.

[52] Wen Y., Pan X.-F., Zhao Z.-M., Chen F., Fu C.-J., Li S.-Q., Zhao Y., Chang H., Xue Q.-P., Yang C.-X. (2014). Knowledge of human papillomavirus (HPV) infection, cervical cancer, and HPV vaccine and its correlates among medical students in Southwest China: A multi-center cross-sectional survey. Asian Pac. J. Cancer Prev..

[53] Blödt S., Holmberg C., Müller-Nordhorn J., Rieckmann N. (2012). Human papillomavirus awareness, knowledge and vaccine acceptance: A survey among 18–25 year old male and female vocational school students in Berlin, Germany. Eur. J. Public Health.

[54] Marlow L.A.V., Zimet G.D., McCaffery K.J., Ostini R., Waller J. (2013). Knowledge of human papillomavirus (HPV) and HPV vaccination: An international comparison. Vaccine.

[55] Rančić N.K., Golubović M.B., Ilić M.V., Ignjatović A.S., Živadinović R.M., Đenić S.N., Momčilović S.D., Kocić B.N., Milošević Z.G., Otašević S.A. (2020). Knowledge about cervical cancer and awareness of human papillomavirus (HPV) and HPV vaccine among female students from Serbia. Medicina.

[56] López N., Garcés-Sánchez M., Belén Panizo M., Salamanca de la Cueva I., Artés M.T., Ramos B., Cotarelo M. (2020). HPV knowledge and vaccine acceptance among European adolescents and their parents: A systematic literature review. Public Health Rev..

[57] Public Health Scotland A Guide to the HPV Vaccine. http://www.healthscotland.com/documents/5988.aspx.

[58] Loke A.Y., Kwan M.L., Wong Y.-T., Wong A.K.Y. (2017). The uptake of human papillomavirus vaccination and its associated factors among adolescents: A systematic review. J. Prim. Care Community Health.

[59] Rodriguez A.M., Do T.Q.N., Goodman M., Schmeler K.M., Kaul S., Kuo Y.-F. (2019). Human papillomavirus vaccine interventions in the U.S.: A systematic review and meta-analysis. Am. J. Prev. Med..

[60] Radisic G., Chapman J., Flight I., Wilson C. (2017). Factors associated with parents’ attitudes to the HPV vaccination of their adolescent sons: A systematic review. Prev. Med..

[61] Marlow L.A.V., Chorley A.J., Haddrell J., Ferrer R., Waller J. (2017). Understanding the heterogeneity of cervical cancer screening non-participants: Data from a national sample of British women. Eur. J. Cancer.

[62] Paulauskiene J., Ivanauskiene R., Skrodeniene E., Petkeviciene J. (2019). Organised versus opportunistic cervical cancer screening in urban and rural regions of Lithuania. Medicina.

[63] World Health Organization World Health Assembly Adopts Global Strategy to Accelerate Cervical Cancer Elimination. https://www.who.int/news/item/19-08-2020-world-health-assembly-adopts-global-strategy-to-accelerate-cervical-cancer-elimination.

