# Supplementary material for: Students’ Knowledge about Cervical Cancer Prevention in Poland
Source: Medicina (Kaunas). 2021 Sep 30;57(10):1045. doi: 10.3390/medicina57101045 (PMC8539101; doi:10.3390/medicina57101045)
Supplement: Supplementary file 1 [file medicina-57-01045-s001.zip › medicina-1375125-supplementary.pdf]

THE STUDENTS' KNOWLEDGE ABOUT THE PREVENTION OF CERVICAL CANCER

1. What is the main factor risk of cervical cancer?
a) Smoking cigarettes
b) Early initiation of sexual intercourse/many sexual partners
c) Human papillomaviruses (HPV)
d) Chlamydiosis

2. Is cervical cancer genetically inherited?
a) Yes
b) No

3. What is the characteristic symptom of early stage cervical cancer?
a) Bleeding beyond menstruation
b) Abundant vaginal discharge
c) Pain during sexual intercourse
d) There are no characteristic symptoms of early stage cervical cancer

4. What is the name of the screening test for cervical cancer?
a) Cystoscopy
b) Pap test
c) Colposcopy
d) Vaginal ultrasound

5. What is the screening test for cervical cancer?
a) Examination of the cervix using an optical equipment
b) Gynecological examination of the cervix
c) Microscopic evaluation of the exfoliated cells from the vaginal part of the cervix
d) Biopsy of the cervix

6. When should the first prophylactic test for cervical cancer be performed?
a) Shortly after sexual initiation
b) Before a planned pregnancy
c) During the first pregnancy
d) After the birth of the first child

7. To women, at what age is the Population Program for the Prevention and Early Detection of Cervical Cancer in Poland directed?
a) Women from 18 years old every year
b) Women 25-59 years old every year
c) Women 25-59 years old every 3 years
d) Women 35-69 years old every 3 years

8. What is the most important for reducing the own risk of cervical cancer?
a) Use of a condom
b) Not using hormonal contraception
c) Eating healthy and being physically active
d) Vaccination against HPV viruses

Gender:

- o Woman
- o Man

Age: ………………. years

Faculty:

- o Medicine
- o Health Sciences
- o Veterinary Medicine
- o Technical Sciences
- o Law/Administration
- o Art
- o Humanistic
- o Social Sciences
- o Theology
- o Other

Degree of study:

- o Bachelor's degree
- o Master degree

Are you sexually active?

- o Yes
- o No

Have you ever had a conversation with someone about cervical cancer?

- o Yes with who?..
- o No
